# Microbiological Diagnosis: A Key to Successfully Managing Cryptococcal Osteomyelitis Coexisting With Leprosy

**DOI:** 10.7759/cureus.80143

**Published:** 2025-03-06

**Authors:** Archana Keche, Sushree Sarathi, Soma Jana, Yashita Gupta

**Affiliations:** 1 Microbiology, All India Institute of Medical Sciences, Raipur, Raipur, IND; 2 Pathology and Laboratory Medicine, All India Institute of Medical Sciences, Raipur, Raipur, IND

**Keywords:** cryptococcal osteomyelitis, cryptococcus neoformans infection, cutaneous, fungal infection, lepromatous leprosy

## Abstract

*Cryptococcus neoformans*, an encapsulated yeast-like fungus, can induce a varied array of symptoms like meningitis, cryptococcomas of the brain, spinal cord granuloma, and disseminated disease, more so in people with impaired immune systems. Nonetheless, osseous lesions are rare. A 27-year-old male presented with a painful ulcer on his left lateral ankle. His medical history revealed leprosy for the last three years. The patient underwent an incisional biopsy and surgical debridement, and both the samples grew *Cryptococcus neoformans* on culture. Cryptococcal osteomyelitis involving the calcaneus and tibia was established. The patient was treated with liposomal amphotericin B followed by fluconazole.

Cryptococcal osteomyelitis, though uncommon, should be included in the differential diagnosis of chronic osteolytic lesions for optimal patient care. This case highlighted that early diagnosis and confirmation through microbiological investigations are crucial for the successful management of these rare and potentially debilitating infections, even in immunocompetent individuals.

## Introduction

Cryptococcal osteomyelitis is a rare manifestation of *Cryptococcus neoformans* infection, typically seen in immunocompromised individuals, such as those with HIV/AIDS, organ transplant recipients, or those undergoing immunosuppressive therapy [1]. It is primarily acquired through the inhalation of environmental spores and can disseminate hematogenously to other organs, including the brain, skin, and bones [2]. While* C. neoformans* most commonly causes meningeal and pulmonary infections, skeletal involvement is unusual due to the limited blood flow to bones and the relatively low frequency of hematogenous dissemination to the musculoskeletal system, and it often presents diagnostic challenges [3]. When bone involvement does occur, the commonest sites are ribs, vertebrae, and femur.

This case report describes the presentation, diagnosis, and management of cryptococcal osteomyelitis in a 27-year-old male with a history of multibacillary lepromatous leprosy. The patient, who had been treated with immunosuppressive therapies for erythema nodosum leprosum (ENL), developed a painful ulcer on the left ankle, which was ultimately diagnosed as cryptococcal osteomyelitis after a thorough microbiological and histopathological evaluation.

Immune dysregulation and repeated use of immunosuppressive therapies, such as thalidomide and corticosteroids, to manage reactions like ENL in leprosy increase susceptibility to opportunistic infections, including cryptococcosis. This case highlights the critical role of microbiological investigations in the timely diagnosis and successful treatment of rare infections, particularly in patients with complex medical histories.

## Case presentation

A 27-year-old patient presented to our Outpatient Department (OPD) with complaints of painful ulcers over his left heel for three months. There was no history of accidental skin trauma. His medical history revealed that he had had multibacillary (MB) lepromatous leprosy for three years and had completed his MB-multidrug therapy, including dapsone, rifampicin, and clofazimine. Later on, he developed several episodes of ENL and was on thalidomide and steroids on and off for three years.

**Figure 1:**
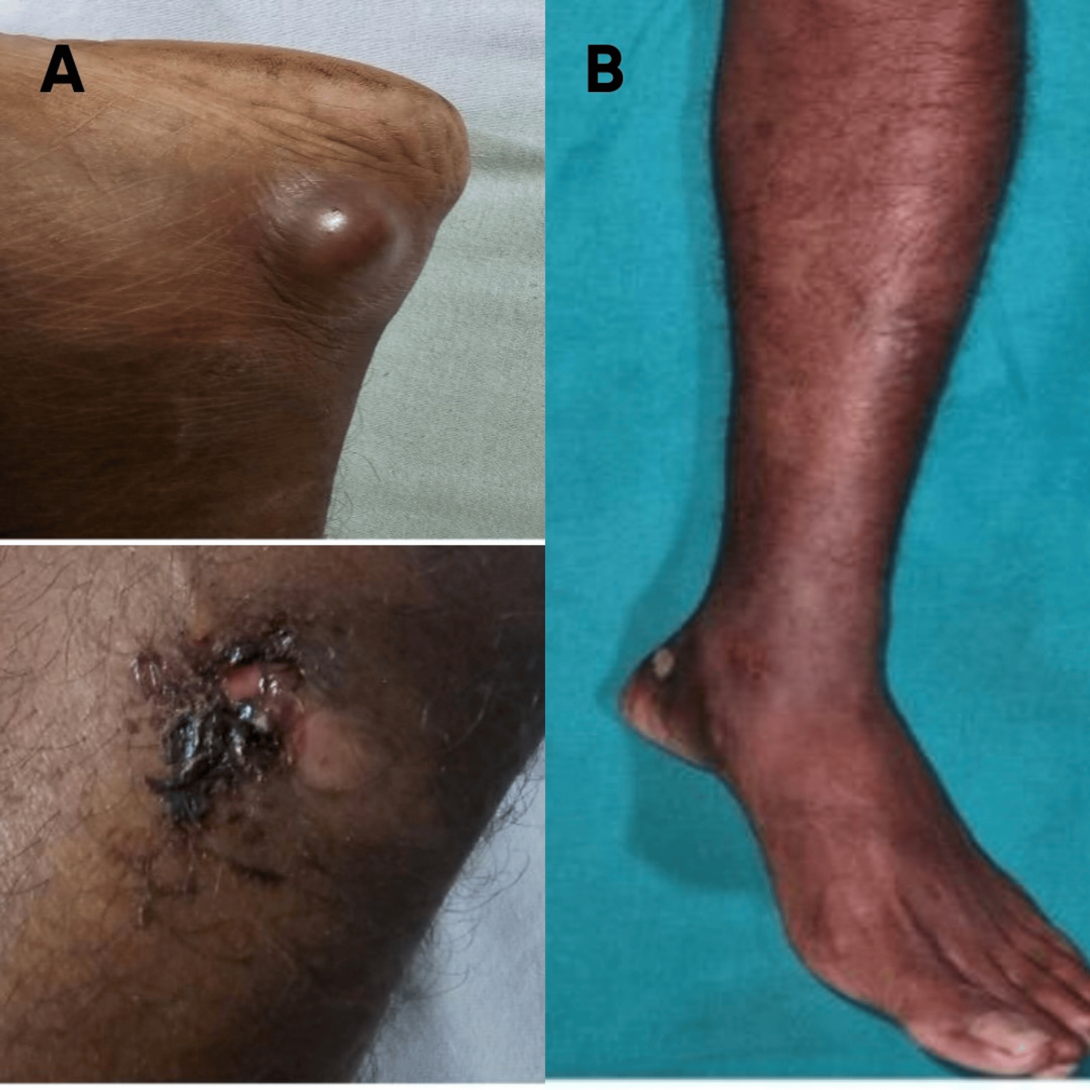
Clinical images A: cutaneous lesions before treatment; B: healed lesions after treatment

To begin with, there was a cystic swelling on his left heel, which gradually increased in size and later ruptured to form a painful ulcer. Upon initial hospital visit, physical examination revealed a major ulcer (3x3) with irregular and raised margins, located two centimeters below the medial malleolus. There was a core loss of material and three other minor ulcers over the ankle. Erythema and edema were present on the skin surrounding the ulcers, and purulent fluid was exuded with pressure (Figure 1A). The joint examination was also significant for a decreased range of ankle movements. There were no systemic symptoms, no further skin involvement, and no regional lymphadenopathy. The patient denied having recently traveled and had no history of obvious contact with pigeons, bird droppings, or other animals.

**Figure 2:**
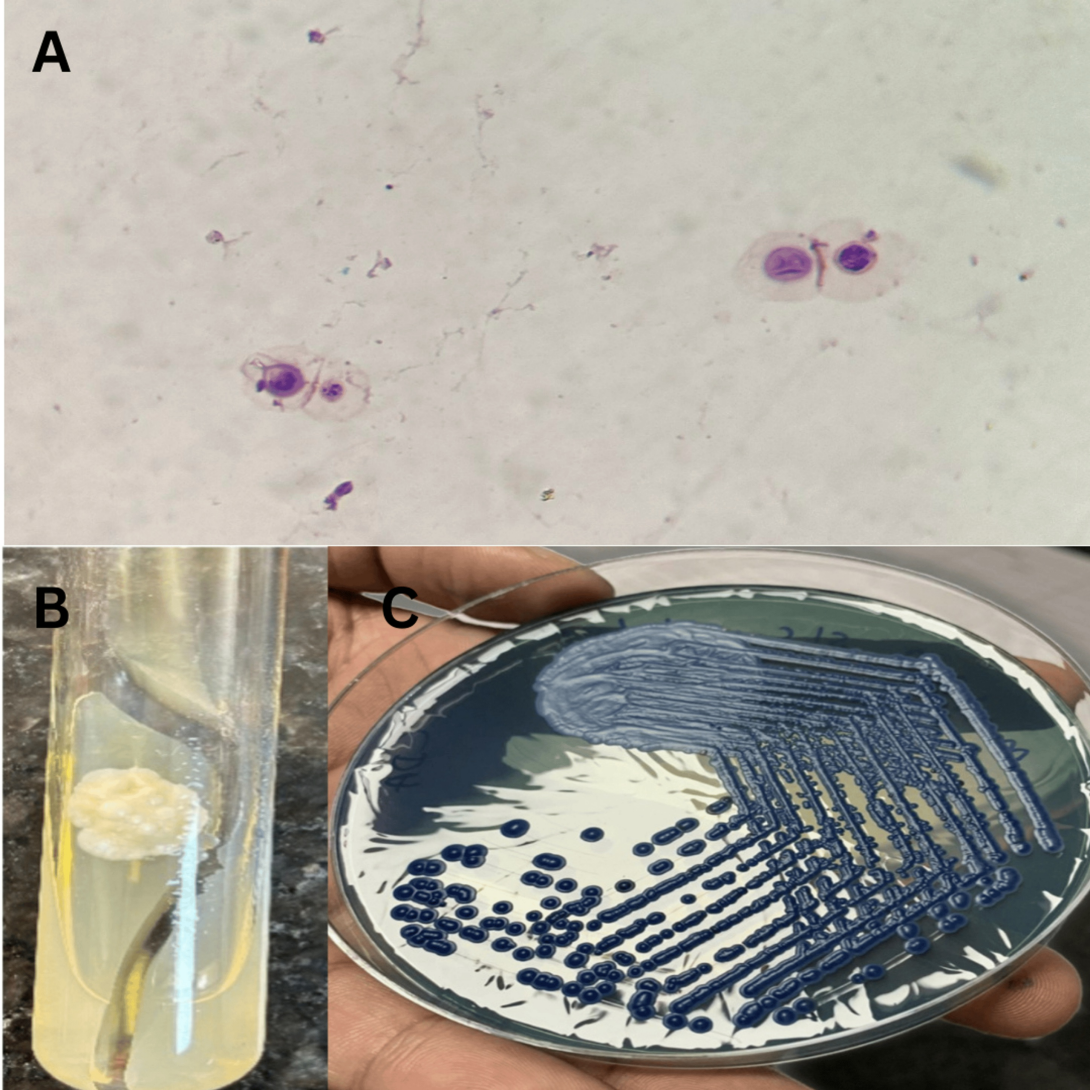
Morphology of Cryptococcus neoformans A: Gram stain of FNAC sample (100x) showing encapsulated, round yeasts, with some budding forms; B: white smooth colonies on Sabouraud’s Dextrose Agar; C: light blue, dry colonies on *Cryptococcus* differential agar after incubation at 37°C FNAC: fine needle aspiration cytology

Blood tests revealed the C-reactive protein (CRP) level to be 1.4 mg/L (reference range <10 mg/L), and the total leukocyte count was elevated at 14.16 x 103/uL (reference range: 4-10 x 103/uL) with a normal lymphocyte count. HIV serology came out negative. The initial evaluation suggested several conditions in the differential diagnosis, including bacterial osteomyelitis, tuberculosis (TB) infection, and diabetic foot ulcers. However, the absence of systemic symptoms such as fever and normal CRP made a bacterial infection less likely. The absence of a history of diabetes, along with the lack of significant ischemia or neuropathy, ruled out diabetic foot ulcers. Radiological investigations of the chest and abdomen did not indicate any signs of systemic TB involvement. Pus was received for fungal and bacterial cultures in the Department of Microbiology. Numerous mucoid colonies were observed on Sabouraud Dextrose Agar + Chloramphenicol after 48 hours of incubation, and these colonies were identified as *Cryptococcus neoformans *(Figures 2A, 2B). Further confirmation was done by a positive rapid urease test, brown pigmented colonies on bird-seed agar, and the VITEK 2 (bioMérieux, Inc., Marcy-l'Étoile, France) compact identification system. C. gatti was ruled out by the luxuriant growth on the Cryptococcal differential agar (Figure 2C).

In addition, a lesion biopsy was taken for a histological and cultural analysis, which confirmed the presence of *C. neoformans. *Additional tests, including chest X-rays, abdominal ultrasonography, and blood cultures, were performed in order to rule out a systemic cryptococcal infection. All of these tests produced negative results. A lumbar puncture was not performed as he did not exhibit any neurological symptoms.

**Figure 3:**
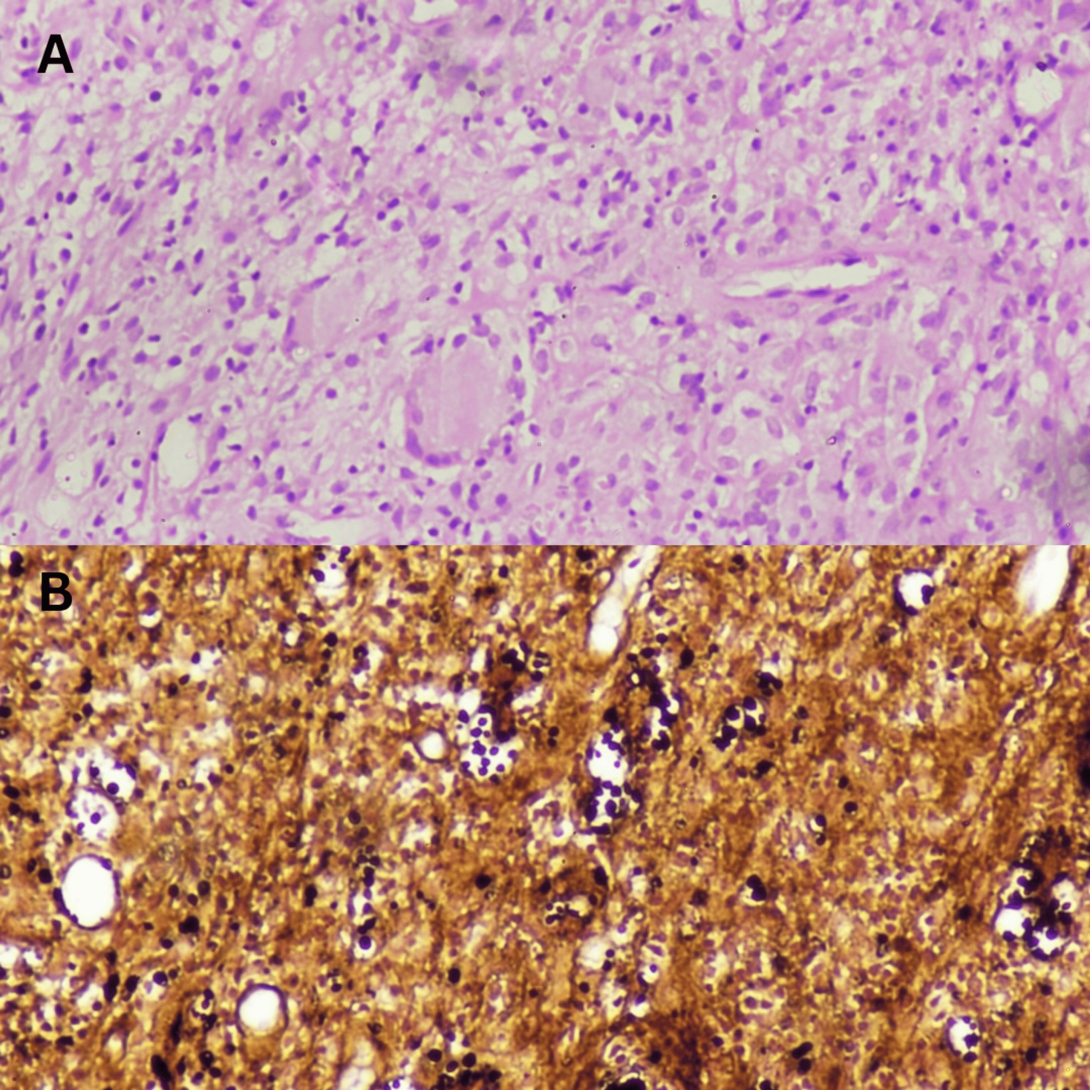
Histopathology images A: H&E (10X) image shows dense mixed inflammatory infiltrate along with ill-defined collections of epithelioid histiocytes and numerous multinucleated giant cells; B: GMS (10X) highlight round to oval encapsulated yeasts with thin cell walls suggestive of *Cryptococcus spp.* GMS: Grocott-Gömöri's methenamine silver stain; H&E: hematoxylin and eosin

An MRI of his left lower extremity revealed an ill-defined altered signal intensity in the calcaneum with restricted diffusion in the center of the collection and peripheral rim enhancement, which was suggestive of a calcaneal abscess. Furthermore, there was short tau inversion recovery (STIR) hyperintensity in the tibial head and soft tissue invasion. Following the imaging, the patient underwent wound debridement and joint irrigation of his left ankle. The tissue was sent for histopathological examination (HPE) and culture. HPE revealed dense mixed inflammatory cell infiltrate suggestive of infective etiology, whereas Grocott-Gömöri's methenamine silver stain (GMS) revealed round to oval encapsulated yeasts with thin cell walls (Figures 3A, 3B). On the third day, a few yeasts were seen on culture, which were identified as *Cryptococcus neoformans*, after which the diagnosis was established as cryptococcal osteomyelitis. Intravenous liposomal amphotericin B 3 mg/kg daily was started. The patient's overall health and apyretic state persisted. During the monthly follow-up, the skin lesion gradually improved, and routine blood tests revealed no fluctuations. When the skin ulcers and indications of skin inflammation fully disappeared after three months, he was switched to a long-term regimen of oral fluconazole 400 mg/day. In the following six months, the patient was in contact telephonically. There was no indication of a recurrence (Figure 1B).

## Discussion

Cryptococcal infection is still linked to a high death rate despite breakthroughs in diagnosis and treatment because of a lack of understanding regarding clinical features and risk factors. The greatest risk of dissemination to almost any organ, but particularly the central nervous system, is present in patients with significant qualitative or quantitative T cell anomalies [4]. Cryptococcal osteomyelitis is an unusual symptom of cryptococcal infection, and it most commonly affects the ribs, vertebrae, and femur. The involvement of the ankle joint, as seen in our patient, is rare [5].

In this article, we describe the effective management of ankle joint cryptococcal osteomyelitis. This is the only case of cryptococcal osteomyelitis in a leprosy patient that has been documented in the literature, as far as we are aware. Our patient, however, was HIV-negative and had no other predisposing factors typically associated with cryptococcal infections, such as recent travel to endemic areas or exposure to birds. We think that the fact that our patient had been taking thalidomide and corticosteroids for a long time because of his ENL reaction made him more likely to get the disease.

Patients typically experience painful soft tissue swelling that progresses over time, along with systemic symptoms like fever [6]. White cell count is usually normal, and the erythrocyte sedimentation rate (ESR) is variably elevated. Our patient showed painful soft tissue swelling and a slightly elevated white cell count. In addition to the rare osteomyelitis we report here, cryptococcal infections have been documented with atypical cutaneous manifestations. For instance, Aggarwal et al. described a case of a pigmented facial nodule in a patient, highlighting the diverse cutaneous presentations of cryptococcosis that may be overlooked or misdiagnosed as other skin lesions or infections, especially in immunocompromised patients [7].

Research data suggest that antifungals (amphotericin B, with or without flucytosine, followed by fluconazole for long-term therapy) combined with surgical debridement can effectively treat cryptococcal osteomyelitis [8]. The same protocol was followed to treat our patient.

Regarding expected outcomes, cryptococcal osteomyelitis, when diagnosed and treated early, generally carries a favorable prognosis, especially when managed with a combination of antifungal therapy and surgical debridement. However, recurrence rates in these cases vary and are often influenced by the patient’s immune status and adherence to long-term therapy. Our patient remains asymptomatic at this point, and there was no evidence of recurrence during the one year of monitoring. The recurrence rate in cryptococcal osteomyelitis can be higher in cases involving more severe immunosuppression, especially when therapy is incomplete or when other underlying conditions are present.

The leprosy case having nodular cutaneous lesions with an atypical presentation with multiple differential diagnoses, simple microscopy, insistent follow-up, and isolation of *Cryptococcus* from a calcaneal abscess provided a definitive diagnosis of cryptococcal osteomyelitis. Microbiological diagnosis of incidental detection of *Cryptococcus neoformans* proved a successful key for the management of the patient.

## Conclusions

To conclude, the case presented with a cystic lesion on the left lower extremities, leading to a complex diagnostic workup as a rare presentation with a history of leprosy and intake of immunosuppressant therapy. While initial investigations suggested other possibilities, microscopic examination and fungal culture revealed cutaneous cryptococcosis. Further evaluation with insistent follow-up identified cryptococcal osteomyelitis in the calcaneus and tibia, which was confirmed by isolating *Cryptococcus neoformans* from aspirate. Despite the favorable outcome in this case, clinicians should be aware that the recurrence of cryptococcal osteomyelitis is possible, particularly in patients with ongoing immunosuppression. Long-term follow-up is essential to monitor for signs of recurrence and to ensure adherence to antifungal therapy. This case serves as a reminder of the need for vigilant long-term management in patients with a history of immunosuppressive treatment, as the risk of opportunistic infections may remain even after the resolution of the primary disease. Cryptococcal osteomyelitis remains a rare complication, even in patients with compromised immune systems. This case serves as a reminder that such rare manifestations can occur in immunocompetent or partially immunocompromised patients, emphasizing the importance of maintaining a broad differential diagnosis. The combination of surgical debridement and antifungal therapy contributed significantly to the successful management of this rare infection.

This case also highlights the importance of meticulous microbiological evaluation, even for incidental findings, as it proved crucial for a definitive diagnosis and effective management of the patient.
